# Thin-film metallic glass: an effective diffusion barrier for Se-doped AgSbTe_2_ thermoelectric modules

**DOI:** 10.1038/srep45177

**Published:** 2017-03-22

**Authors:** Chia-Chi Yu, Hsin-jay Wu, Ping-Yuan Deng, Matthias T. Agne, G. Jeffrey Snyder, Jinn P. Chu

**Affiliations:** 1 Department of Materials Science and Engineering, National Taiwan University of Science and Technology, Taipei 10607, Taiwan; 2 Department of Materials and Optoelectronic science, National Sun Yat-sen University, Kaohsiung 80424, Taiwan; 3 Department of Materials Science and Engineering, Northwestern University, Evanston, IL 60208, USA

## Abstract

The thermal stability of joints in thermoelectric (TE) modules, which are degraded during interdiffusion between the TE material and the contacting metal, needs to be addressed in order to utilize TE technology for competitive, sustainable energy applications. Herein, we deposit a 200 nm-thick Zr-based thin-film metallic glass (TFMG), which acts as an effective diffusion barrier layer with low electrical contact resistivity, on a high-*zT* Se-doped AgSbTe_2_ substrate. The reaction couples structured with TFMG/TE are annealed at 673 K for 8–360 hours and analyzed by electron microscopy. No observable IMCs (intermetallic compounds) are formed at the TFMG/TE interface, suggesting the effective inhibition of atomic diffusion that may be attributed to the grain-boundary-free structure of TFMG. The minor amount of Se acts as a tracer species, and a homogeneous Se-rich region is found nearing the TFMG/TE interface, which guarantees satisfactory bonding at the joint. The diffusion of Se, which has the smallest atomic volume of all the elements from the TE substrate, is found to follow Fick’s second law. The calculated diffusivity (*D*) of Se in TFMG falls in the range of D~10^−20^–10^−23^(m^2^/s), which is 10^6^~10^7^ and 10^12^~10^13^ times smaller than those of Ni [10^−14^–10^−17^(m^2^/s)] and Cu [10^−8^–10^−11^(m^2^/s)] in Bi_2_Te_3_, respectively.

Thermoelectric (TE) materials and modules, which feature the capability of converting waste heat into electricity through the solid-state Seebeck effect, have offered a prospective path to preserve natural resources and to further technological development^1^,^2^,^3^. The thermal-to-electrical conversion efficiency of TE materials is related to the dimensionless figure-of-merit, *zT = (S*^*2*^*/ρκ)T*, where *S* is the Seebeck coefficient, *ρ* represents the electrical resistivity and *κ* stands for the thermal conductivity.

In general, the reduction of κ, which may be achieved by introducing nanoparticles/precipitates in the grains or along the grain boundaries^4^,^5^,^6^, and enhancement of the power factor (PF = *S*^*2*^*/ρ*), which might be realized by optimizing the carrier concentration via doping^7^, could lead to higher *zT*. Despite the large amount of effort to improve *zT*, the utilization of TE modules as a cost-effective and high-efficiency technology has yet to be realized. In working towards this goal, the thermal stability and mechanical strength of the various joints in TE modules, which typically undergo large thermal fluctuations, is likely to be one of the key issues^8^,^9^,^10^,^11^,^12^,^13^.

For example, state-of-the-art *p*-type (Bi, Sb)_2_Te_3_-based materials, which exhibit *zT* above unity near room temperature^14^, can be joined to metallic electrodes using Sn-based solders such as Bi-Sn or Sn_3_Ag_0.5_Cu solder^15^. However, due to the low-melting-point of the solder alloy, the TE modules are limited to low operating temperatures. Alternatively, a solid-liquid interdiffusion bond that results from the incorporation of low-melting point metals/alloys, such as Sn-based or In-based thin films which act as a liquid transient layer^16^, is likely to improve the thermal stability and bonding strength of the joints through the formation of high-temperature intermetallic compounds (IMCs) in-between the electrodes and TE materials. However, concerns have been raised about whether the IMCs in multi-layer interdiffusion bonds would be electrically conductive and exhibit a coefficient of thermal expansion (CTE) similar to the other low-temperature liquid transient layers, the TE materials and the electrodes^17^. Therefore, in order to obtain the maximum electrical power output and module efficiency, a diffusion barrier layer having thin thickness, high thermal stability, and good structural/chemical compatibility with adjacent materials will ensure low electrical and thermal contact resistances at the joint.

For TE modules that operate at temperatures higher than 600 K, the technique of brazing, which introduces a high-melting-point filler metal, is viewed as a possible bonding method, assuming that the thermal stresses accumulated from high-temperature operation and cracking at the brazing joints can be avoided^18^. Consequently, the search for adequate diffusion barrier layers is essential for the above-mentioned bonding methods, in order to inhibit the serious inter-diffusion in-between the solder alloys/filler metals and TE materials. Several studies of the interfacial evolutions of TE/metal joints suggest that although the crystalline metals, such as Ni^19^, Ti^20^,^21^,^22^ or Mo^19^, may seem promising candidates for diffusion barrier layers, their failure is inevitable inside the diffusion barrier layer or at the interface with IMCs after long-term and high-temperature aging^20^,^23^. Taking the CoSb_3_/(Ni, Mo) joints as an example, instead of blocking the interdiffusion between the solder and TE, the Ni diffusion barrier layer dissolves into the solder. Thermal cycling of CoSb_3_/Mo joints results in a fractured interface due to the large CTE mismatch^19^.

Herein, a Zr-based thin-film metallic glass (TFMG) is proposed as an alternative diffusion barrier layer for use in mid-temperature TE modules. For this investigation, Se-doped AgSbTe_2_, which exhibits a high *zT* of *1.4* at ≈673 K^24^, is selected as the TE substrate. The diffusion across the TFMG/TE (Se-doped AgSbTe_2_) interface is evaluated during thermal aging. Due to the lack of a periodic atomic packing structure, TFMG possess superior mechanical properties compared to their crystalline counterparts^25^. Moreover, the grain boundaries and/or crystalline defects in crystalline materials can act as diffusive paths for atoms during thermal annealing. TFMGs, however, exhibit high structural disorder and a grain boundary-free structure, leading to their success as diffusion barriers in copper metallization^26^, CIGS solar cells^27^ and tin whisker mitigation of microelectronic packaging^28^, despite the short-term annealing of these systems. As a result of having an amorphous structure, the thermal conductivity of ZrCuAlNi metallic glass, reported to be ~5 W/mK at 300 K^29^, is lower than that of typical metallic electrodes (e.g. Ni and Ti, *κ* > 20 W/mK at 300 K). Nevertheless, the influence of thermal resistance of TFMG can be ignored due to the thin thickness (~200 nm) of TFMG (compared to mm-thick TE leg).

In order to evaluate the feasibility of TFMG as a diffusion barrier, a 200-nm-thick ZrCuAlNi TFMG layer is deposited on one side of the Ag_25_Sb_25_Se_5_Te_45_ (TE substrate), while a crystalline Ni layer with the same thickness is deposited on the other side of the TE substrate, to form a sandwich-like structure of TFMG (200 nm)/TE substrate/Ni (200 nm). After thermal aging from 8 to 360 h at 673 K, the Se atom, originating from the TE substrate, diffuses into the TFMG and forms a homogeneous Se-rich region nearing the TFMG/TE interface, which ensures satisfactory bonding at the joint. Furthermore, no IMCs are found at the interface between the TFMG and TE substrate, reducing the possibility of high electrical contact resistance and low ductility that is commonly observed in electronic packages^30^. For example, the electrical resistivity has been observed to increase with the annealing time in Cu/Sn-3Cu/Cu reaction couples^30^, mainly attributed to the increasing thickness of the IMC reaction layer.

Contrary to the TFMG/TE result, the diffusion couple of Ni/TE reveals extensive formation of Ni-Te-Sb IMCs and voids even after comparably short-term annealing. This suggests that the amorphous thin film, which provides less channels for diffusion than that of a (poly)crystalline one, might be appropriate for use as a diffusion barrier layer in mid-temperature TE modules.

## Results and Discussion

**Figure 1:**
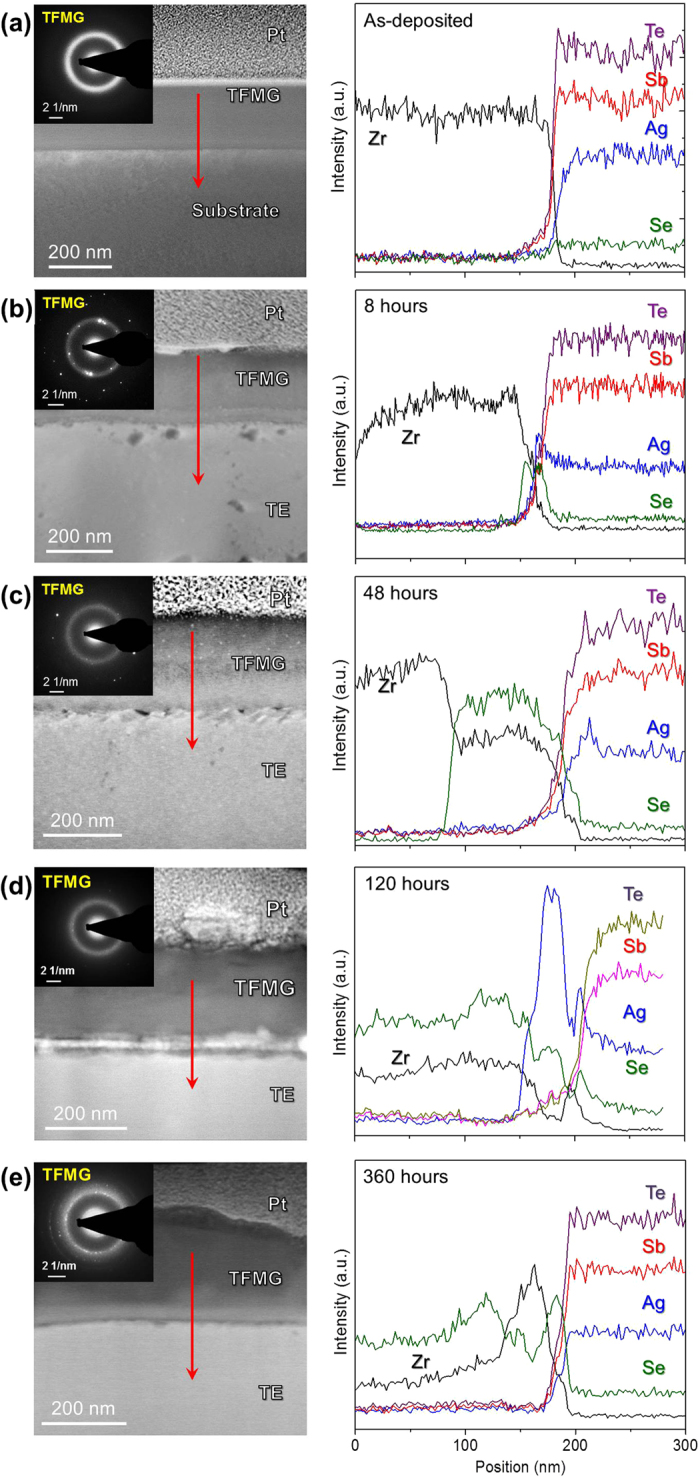
Cross-sectional STEM images together with the elemental line scan results of TFMG/AgSbSe_x_Te_2-x_ couples in (**a**) as-deposited condition and reacted at 673 K for (**b**) 8 hours, (**c**) 48 hours (**d**) 120 hours and (**e**) 360 hours. All the diffraction patterns embedded in the upper-left corners were obtained from the TFMG layer.

The thermal properties of the TFMG were characterized using DSC. The glass transition temperature, *T*_*g*,_ and crystallization temperature, *T*_*x*_, of Zr_60_Cu_24_Al_11_Ni_5_ TFMG were determined to be 682.9 K (409.9 °C) and 770.6 K (497.6 °C), respectively (see Supplementary Figure S1). The thermal aging of TFMG/TE couples was carried out below *T*_*g*_ to ensure no crystallization occurs. The wide supercooled liquid region (∆*T*_*x*_ = *T*_*x*_ −* T*_*g*_) of TFMG implies a higher resistance to the nucleation and growth of crystalline phases upon annealing^31^. After long-term annealing, the TFMG remained well-adhered to the TE surface. The low magnification top-view SEM image of the TFMG/TE sample annealed for 24 hours shows no sign of spallation, buckling or incomplete coverage of the TFMG (Supplementary Figure S2). In addition to high thermal stability, a low electrical contact resistivity (ρ_C_) is desirable in TFMG/TE couples. In TE devices, the ρ_C_ between the TE material and metal electrode is expected to fall in the range of 10^−9^ (Ω m^2^) to 10^−8^ (Ω m^2^)^32^. The ρ_C_ between the TFMG and TE substrate, obtained using the transmission line measurement (TLM) method (Supplementary Figure S3), clearly satisfies the requirement having a value of 1.62 × 10^−9^ (Ω m^2^). The electrical properties, including sheet resistance, resistivity, contact resistance and specific contact resistivity of TFMG/Si, Ni/Si, and TFMG/TE are further summarized in Supplementary Table S1. Typical cross-sectional STEM images, SAED patterns and EDS line-scan results of as-deposited TFMG/TE samples and those annealed at 400 °C for 8–360 h are shown in Fig. 1(a–e). The red arrows in the images indicate the EDS line-scan direction. The corresponding compositional profiles are presented to the right of the images. In the EDS line-scan results, the Zr signal represents the TFMG. The SAED patterns of the TFMG layers are embedded in each micrograph. In Fig. 1(a), the featureless amorphous structure and a typical halo SAED pattern of TFMG is shown in the as-deposited condition. After annealing at 673 K for 8 h there was no indication that IMCs had formed, while a homogeneous diffusion layer with a thickness of ≈30 nm was found in the TFMG coating [Fig. 1(b)]. The compositional profile indicates this layer is Se-rich. SAED of the TFMG layer exhibits a halo pattern with minor diffraction-spots, which may be due to oxidation-induced-crystallization in the TFMG upon annealing. After annealing for 48 h [Fig. 1(c)], the width of the Se-rich diffusion layer was found to homogeneously increase to ≈110 nm. The Se diffusion layer continued to grow with increasing annealing time. Se atoms were found to have diffused throughout the entire TMFG layer after 120 h [Fig. 1(d)] and 360 h [Fig. 1(e)]. From the SAED pattern, we found that the main phase of the whole TFMG layer remained amorphous after annealing for 8–120 h. This indicates that Se atoms, diffusing into TFMG, do not react with TFMG to form a crystalline phase. Additionally, the TFMG layer did not react with the TE substrate to form detrimental IMCs. In brief, for every annealed TFMG/TE sample, there was a well-defined, continuous interface.

**Figure 2:**
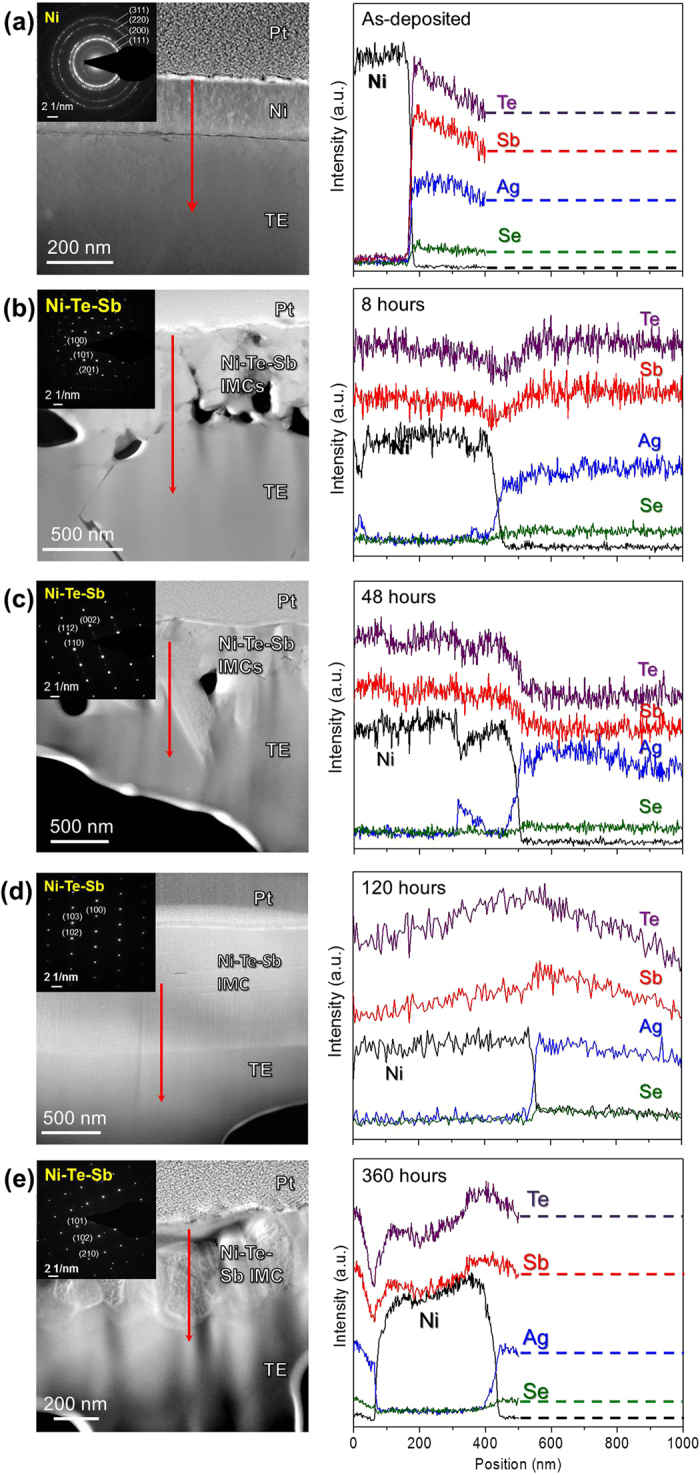
Cross-sectional STEM images together with the elemental line scan results of Ni/AgSbSe_x_Te_2-x_ couples in (**a**) as-deposited condition and reacted at 673 K for (**b**) 8 hours, (**c**) 48 hours (**d**) 120 hours and (**e**) 360 hours. The diffraction patterns embedded in the upper-left corners were obtained from Ni layer in (**a**) and Ni-Te-Sb IMCs regions in (**b–e**).

The cross-sectional STEM images and EDS line-scan results of as-deposited, 8-, 48-, 120-, and 360-hours-annealed Ni/AgSbSe_x_Te_2-x_ samples can be found in Fig. 2(a–e). In the as-deposited condition, the 200 nm thick polycrystalline Ni layer and the clearly defined film/substrate interface were observed [Fig. 2(a)]. The ring SAED pattern obtained from the Ni layer exhibited face-centered cubic structure, as identified from the (111), (200), (220) and (311) planes [see the inset of Fig. 2(a)]. In all annealed samples [Fig. 2(b–e)], the entire Ni layer significantly reacted with the TE substrate to form IMCs. The reaction layer thickness was larger than 500 nm. The SAED patterns, acquired from a single grain in the reaction layer, and EDS investigations suggest that hexagonal NiSb_1-x_Te_2x_ (0.28 < × < 0.66) is the primary IMC formed from annealing at 673 K. We note that NiSb_1-x_Te_2x_ is an equilibrium phase reported in the Ni-Te-Sb system at 673 K (400 °C)^33^. Due to the variation of grain orientations in the reaction layer, SAED patterns with different zone axes were obtained [see the insets of Fig. 2(b–e)]. Additionally, the pores seen in the images of the Ni/TE samples can be attributed to the preferential etching of more reactive regions at the grain-boundaries of IMCs and at the IMC/TE interface upon FIB milling. Nevertheless, the formation of IMCs in the Ni diffusion barrier can lead to poor mechanical properties at the joint as well as high electrical- and thermal-resistance contacts.

**Figure 3:**
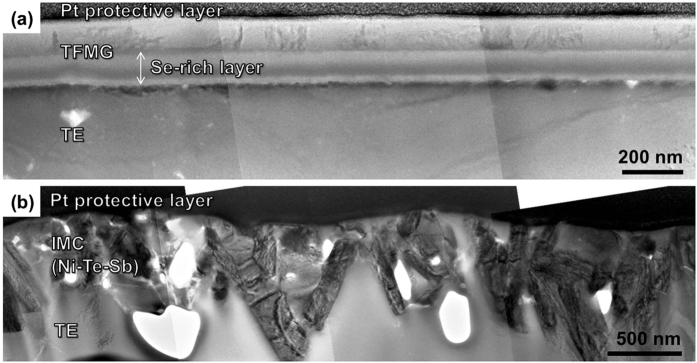
Low magnification bright-field images of (**a**) TFMG/AgSbSe_x_Te_2-x_ and (**b**) Ni/AgSbSe_x_Te_2-x_ couples reacted at 673 K for 48 hours.

The comparison shown in Fig. 3 visually demonstrates that the Zr-based TFMG acted as an effective diffusion barrier layer even after long-term high-temperature annealing, while the Ni layer did not. The extensive interdiffusion/reaction of Ni, Sb and Te resulted in a wide (>500 nm) interfacial reaction zone comprised of NiSb_1-x_Te_2x_ intermetallic compounds and cracks, which could lead to electrical or mechanical failure in thermoelectric modules. However, the success of the TFMG as an effective diffusion barrier may be largely attributed to the formation of a homogeneous Se-rich layer at the TFMG/TE interface (Fig. 3(a)). Having a thickness of ≈100 nm after annealing at 400 °C for 48 h, this layer ensures good adhesion between the TFMG and the TE substrate. Additionally, using the Se atom as a tracer species allowed for the study of the diffusional properties of the metallic glass.

Diffusion in metallic glasses has drawn a great amount of attention due to its technological relevance^34^. Unlike diffusion in crystalline materials, mainly attributed to vacancy and/or interstitial migration trigged by independent random atomic jumps^35^, the mechanism of diffusion in amorphous glass is generally acknowledged to be a collective hopping process^36^. This mechanism may be responsible for the homogeneous Se-rich diffusion layer as observed in Fig. 3(b). One of the popular approaches to explain the atomic mobility in MGs is the free-volume model proposed previously by Spaepen^37^. According to this model, the diffusion defects and flow defects, the so-called free volume, serve as both diffusive and viscous media in amorphous alloys. These free volumes are frozen into the structure upon forming the alloy. Using faster quench rates to fabricate MGs produces more free volume defects, increasing the excess volume of the structure. It was also found that the diffusivity in MGs is correlated with the excess volume present in the as-quenched material^34^. Below T_g_, due to the high quench rate of sputtering deposition^38^, the TFMG can be viewed as a frozen-in liquid. As such, it has a large amount of homogeneously dispersed free volume. Therefore, the diffusion across the TFMG/TE couple can be simply treated as the one-way diffusion of Se into the TFMG. This may also be expected as Se exhibits the smallest atomic volume among all the elements involved in the TFMG/TE couple. The diffusion of Se can be described by Fick’s second law:


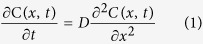


where *C(x, t*) stands for the chemical concentration of the tracer species at a specific position, *x*, and time, *t*, and *D* represents the diffusion coefficient. Given that the diffusion is semi-infinite, the concentration distribution of the tracer species (e.g. Se) follows the thin-film solution of Fick’s second law:


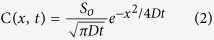


where *S*_*o*_ refers to the initial concentration of tracer atoms (per unit area) at the surface of the as-deposited thin film. Since the annealing time, *t*, was predetermined and the distribution of Se could be obtained from the above-mentioned EDS data, the diffusivity (*D*) of Se was estimated from the slope (−1/4*Dt*) of the logarithmic plot of Eq. (2). Based on the diffusion coefficients reported for various tracer species in metallic glasses^34^, it is expected that D falls in the range of 10^−23^ (m^2^/s) to 10^−16^ (m^2^/s).

**Figure 4:**
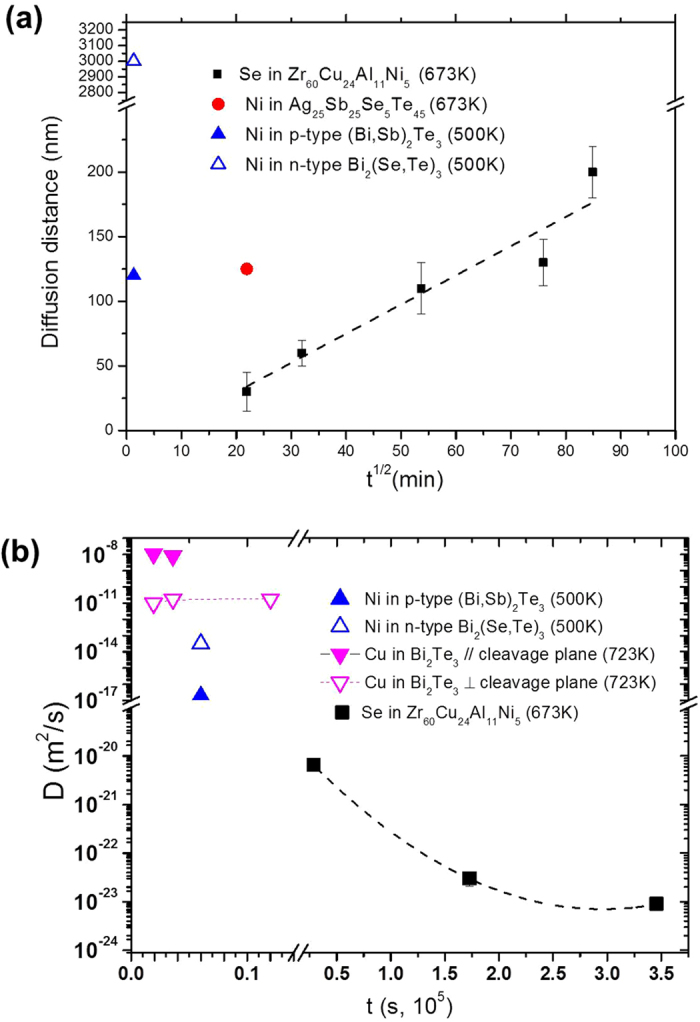
Plots of (**a**) the diffusion distance of Se in TFMG, Ni in TE substrate, Ni in p-type (Bi, Sb)_2_Te_3_ and n-type Bi_2_(Se, Te)_3_[19] vs. the square root of annealing times of 8–120 hours and (**b**) diffusion coefficient (D) of Se atoms in TFMG, Ni in p-type (Bi, Sb)_2_Te_3_ and n-type Bi_2_(Se, Te)_3_[19], Cu in Bi_2_Te_3_ measured parallel and perpendicular to cleavage planes [36] vs. various annealing time.

Figure 4(a) summarizes the Se diffusion distance from the TFMG/TE interface, as determined by the EDS compositional line-scan, for samples annealed 8–120 h at 400 °C. There is a clear square law dependence of the diffusion distance of Se with increasing annealing time. Additionally, after 120 h, the Se was found to be distributed in the whole 200 nm thick TFMG layer. For comparison, the diffusion distance of Ni, determined in this work, and in *p*-type (Bi, Sb)_2_Te_3_ and *n*-type Bi_2_(Se, Te)_3_ at 500 K^39^ are collected in Fig. 4(a). The migration of Ni in crystalline materials is clearly more extensive than that of Se in TFMG. Other determinations about the diffusion of species in crystalline *or* amorphous materials can be deliberated by comparing the diffusion coefficients in Fig. 4(b).

The diffusion coefficient (*D*) of Se in the Zr-based TFMG at 673 K, calculated according to Eq. (2), is shown in Fig. 4(b). The *D* decreases rapidly from D ≈ 6.5 × 10^−21^ (m^2^/s) after 8 h, to a plateau value of *D *≈ 1.2 × 10^−24^ (m^2^/s), after 120 h annealing. This can be ascribed to the structural relaxation of the TFMG during annealing below *T*_*g*_, resulting in the annihilation of excess free volume^40^,^41^, and thus reducing the diffusivity of Se. In other words, atoms require less energy to hop through the less dense structure (as-deposited condition) than to hop through the more dense structure (after annealing). Consequently, the *D* of Se decreases with increasing annealing time as the structure of the TFMG tends from the as-deposited condition towards the fully relaxed state, at which point *D* remains constant [Fig. 4(b)].

The *D* of Ni in *p*-type (Bi, Sb)_2_Te_3_ and *n*-type Bi_2_(Se, Te)_3_ at 500 K^39^ and that of Cu in Bi_2_Te_3_ (measured parallel and perpendicular to the cleavage planes)^42^ are presented together in Fig. 4(b). For further comparison, the *D* of Se in Bi_2_Te_3_ is on the order of 10^−15^–10^−18^ (m^2^/s)^43^ at temperatures along the solidus line. The *D* of Se in amorphous TFMG [≈10^−20^–10^−24^ (m^2^/s)] is 10^6^~10^7^ and 10^12^~10^13^ times smaller compared with that of Ni in Bi_2_Te_3_ [10^−14^(m^2^/s) −10^−17^(m^2^/s)] and that of Cu in Bi_2_Te_3_ [10^−8^(m^2^/s) −10^−11^(m^2^/s)]. As for the self-diffusion and impurity diffusion coefficients reported for other TFMGs around 673 K (*D *≈ 10^−17^–10^−23^ (m^2^/s), as summarized in ref. 30), we find the D of Se to be of the same magnitude, suggesting that the grain-boundary-free structure, effectively prohibits the interdiffusion at the interface of dissimilar materials.

**Figure 5:**
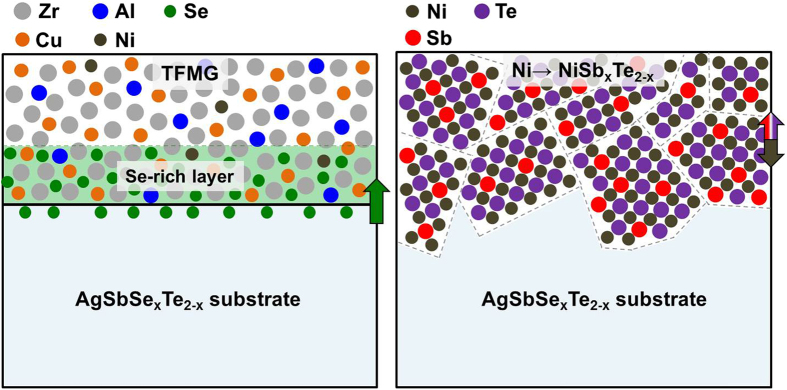
The schematic presentation of the interfacial diffusion of the TFMG/AgSbSe_x_Te_2-x_ and the Ni/AgSbSe_x_Te_2-x_ reaction couples.

Schematic illustrations of the interfacial diffusion of the TFMG/TE and the Ni/TE reaction couples are presented in Fig. 5. Due to the grain boundary-free structure, Se atoms from the TE substrate diffuse uniformly into the TFMG, via its free-volume, during annealing below *T*_*g*_. In contrast, the grain boundaries in the Ni layer serve as an atomic diffusion path, resulting in significant interdiffusion and reaction between Ni and the substrate, so as to form NiSb_x_Te_2-x_ IMCs.

## Conclusion

We demonstrate that the Zr-based TFMG with a thin thickness of 200 nm serves as an effective diffusion barrier layer for mid-temperature TE modules using AgSbTe_2_ as a substrate. After long-term annealing at 673 K, the TFMG/TE interface reveals no observable IMCs, presumably due to the grain-boundary free structure of the TFMG. Instead, a homogeneous Se-rich diffusion region is located at the TFMG/TE interface, indicating good adhesion between the TFMG and the TE substrate. We also find that the diffusion of Se in TFMG follows Fick’s second law while the diffusivity of Se decreases with increasing annealing time, which can be attributed to the annihilation of excess free-volume due to the thermal relaxation of the TFMG. On the other hand, a 200 nm thick Ni layer severely reacts with the TE substrate, under identical annealing conditions, and forms NiSb_x_Te_2-x_ IMCs. As IMCs can be detrimental to the performance of TE modules, the Zr-based TFMG, possessing high strength, high thermal stability and low electrical contact resistivity, shows great potential as an alternative diffusion barrier layer for mid-temperature TE modules.

## Methods

The polycrystalline, quaternary Ag_25_Sb_25_Se_5_Te_45_ alloy, exhibiting *zT* ≈ 1.4 at 673 K^24^, was synthesized by unidirectional solidification in a Bridgman furnace. Details of the Bridgman method have been reported previously^24^. In short, five grams of homogenized Ag_25_Sb_25_Se_5_Te_45_ were loaded in a 7 mm × 9 mm quartz tube, sealed under vacuum and placed in a Bridgman furnace. The three heating zones created a large temperature gradient (50 K/mm) along the axial direction. At first, the ampoule was placed in the high-temperature zone at a temperature above the melting point of the TE alloy (≈1123 K) for at least 1 h, to ensure homogenized melting. Unidirectional solidification was achieved as the ampoule moved gradually downward, at a rate of 5 mm/h, from the high-temperature zone (≈1123 K) to the low-temperature zone (≈300 K). The Bridgman-grown Ag_25_Sb_25_Se_5_Te_45_ was then cut into ~1 mm thick pellets, grinded using a series of SiC sand papers (800–2400 grit), and then polished using Al_2_O_3_ suspension with gradually reduced particle sizes from 1 to 0.05 μm, for the subsequent thin-film deposition process.

Zr_60_Cu_24_Al_11_Ni_5_ (at%) TFMG and pure Ni (99.99%) were deposited on two different sides of the Ag_25_Sb_25_Se_5_Te_45_ substrate by radio-frequency magnetron sputtering with no external heating. Zr_55_Cu_29_Al_11_Ni_5_ alloy and pure Ni sputtering targets were used in a vacuum system with a base pressure of <1 × 10^−6^ Torr. The Ar sputtering pressure was 3 mTorr and a −20 V bias was applied to the substrates for both depositions. After the thin-film depositions, the reaction couple of TFMG/Ag_25_Sb_25_Se_5_Te_45_ (TE substrate)/Ni was again sealed under vacuum and placed in a 673 K furnace for various lengths of time (*i.e.,* 8, 48, 120 and 360 hours).

The sheet resistance and the resistivity were measured using a resistivity meter (Loresta-AX MCP-T370). The contact resistance and specific contact resistance of as-deposited TFMG/TE sample was assessed based on a transmission line measurement^44^ (TLM) technique with the semiconductor parameter analyzer (Agilent B1500A) at room temperature. The TFMG contacts with length of 150 μm and width of 600 μm were deposited on the TE substrate using a shadow mask. The contacts were spaced from 250 to 2000 μm apart. The current (I) and the voltage (V) were measured on the differently spaced contact electrodes and the resistance (*R*) was determined from the inverse slope of a linear fit of the I-V plot.

The thermal properties of the TFMG were characterized using differential scanning calorimetry (DSC, Netzsch 404 F3 Pegasus) from 573 to 973 K (300–700 °C) at a heating rate of 40 K/min under Ar atmosphere. The TFMG layer and TE substrate compositions were determined by energy-dispersive X-ray spectroscopy (EDS) in a scanning electron microscope (SEM), which is part of a dual-beam focused-ion-beam system (FIB, FEI Quanta 3D FEG). Transmission electron microscopy (TEM) sample foils were produced by FIB thinning using 30 kV Ga^+^ beams initially and 5 kV for the final polishing. Prior to FIB thinning, platinum was deposited on top of the samples to protect the thin film-layers from ion-damage. TFMG/TE and Ni/TE microstructures were analyzed by TEM in a FEI Tecnai^TM^ G2 F-20 instrument operated at 200 kV. Selected-area electron diffraction (SAED) with a captured diameter of 200 nm was employed to characterize the structure of local regions in the annealed samples. High-angle annular dark-field (HAADF) imaging and EDS in scanning TEM (STEM) mode were used to acquire the compositional profile across each interface.

## Additional Information

**How to cite this article**: Yu, C.-C. *et al*. Thin-film metallic glass: an effective diffusion barrier for Se-doped AgSbTe_2_ thermoelectric modules. *Sci. Rep.* **7**, 45177; doi: 10.1038/srep45177 (2017).

**Publisher's note:** Springer Nature remains neutral with regard to jurisdictional claims in published maps and institutional affiliations.

## Supplementary Material

Supplementary Information
