# Supplementary material for: Thin-film metallic glass: an effective diffusion barrier for Se-doped AgSbTe2 thermoelectric modules
Source: Sci Rep. 2017 Mar 22;7:45177. doi: 10.1038/srep45177 (PMC5361086; doi:10.1038/srep45177)
Supplement: Supplementary Information [file srep45177-s1.pdf]

Supplementary Information

Thin-film metallic glass: an effective diffusion barrier for Se-doped $AgSbTe_2$ thermoelectric modules

Chia-Chi Yu, Hsin-jay Wu, Ping-Yuan Deng, Matthias T. Agne, G. Jeffrey Snyder[c],

Jinn P. Chu

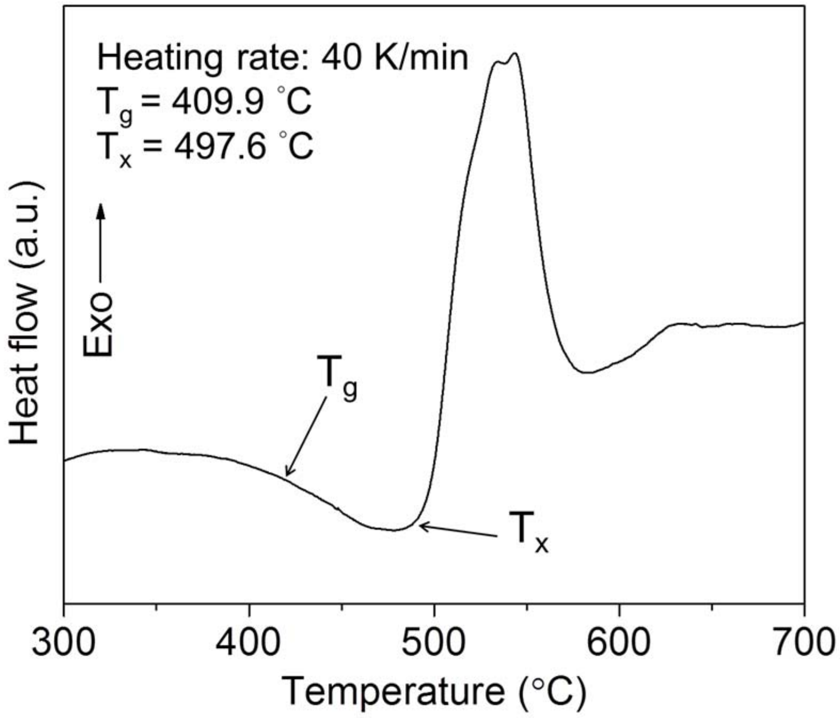

Supplementary Figure S1. Differential scanning calorimetry (DSC) thermogram of an as-deposited $Zr_{60}Cu_{24}Al_{11}Ni_5$ thin film metallic glass. The glass transition temperature, T_g, and crystallization temperature, T_x, were determined to be 682.9 K (409.9 °C) and 770.6 K (497.6 °C), respectively. The supercooled liquid region ($\Delta T_x = T_x - T_g$) of TFMG was 88.3 K.

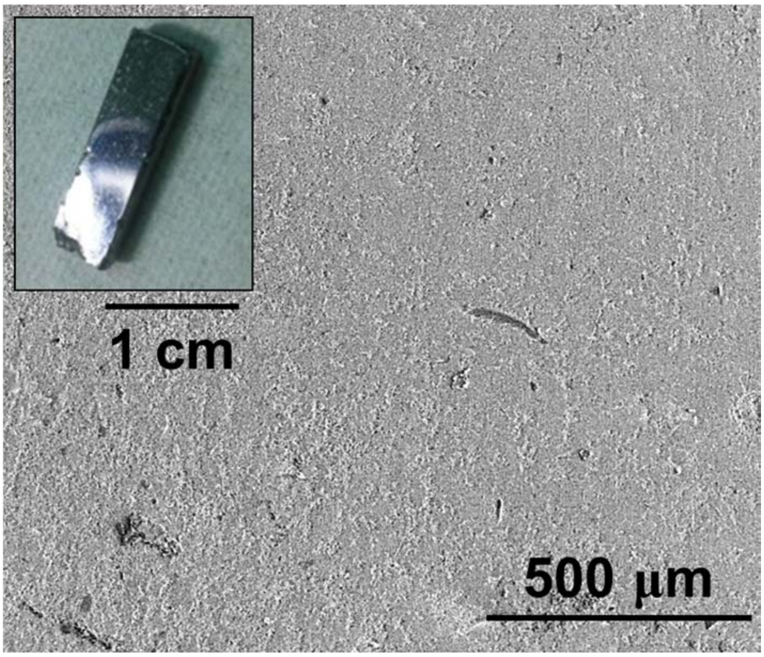

Supplementary Figure S2. Top-View, secondary electrons scanning electron image of TFMG-coated sample, annealed at 673 K for 24 hours, shows good adhesion without observable peeling-off. Inset shows a photograph of the TFMG-coated TE leg.

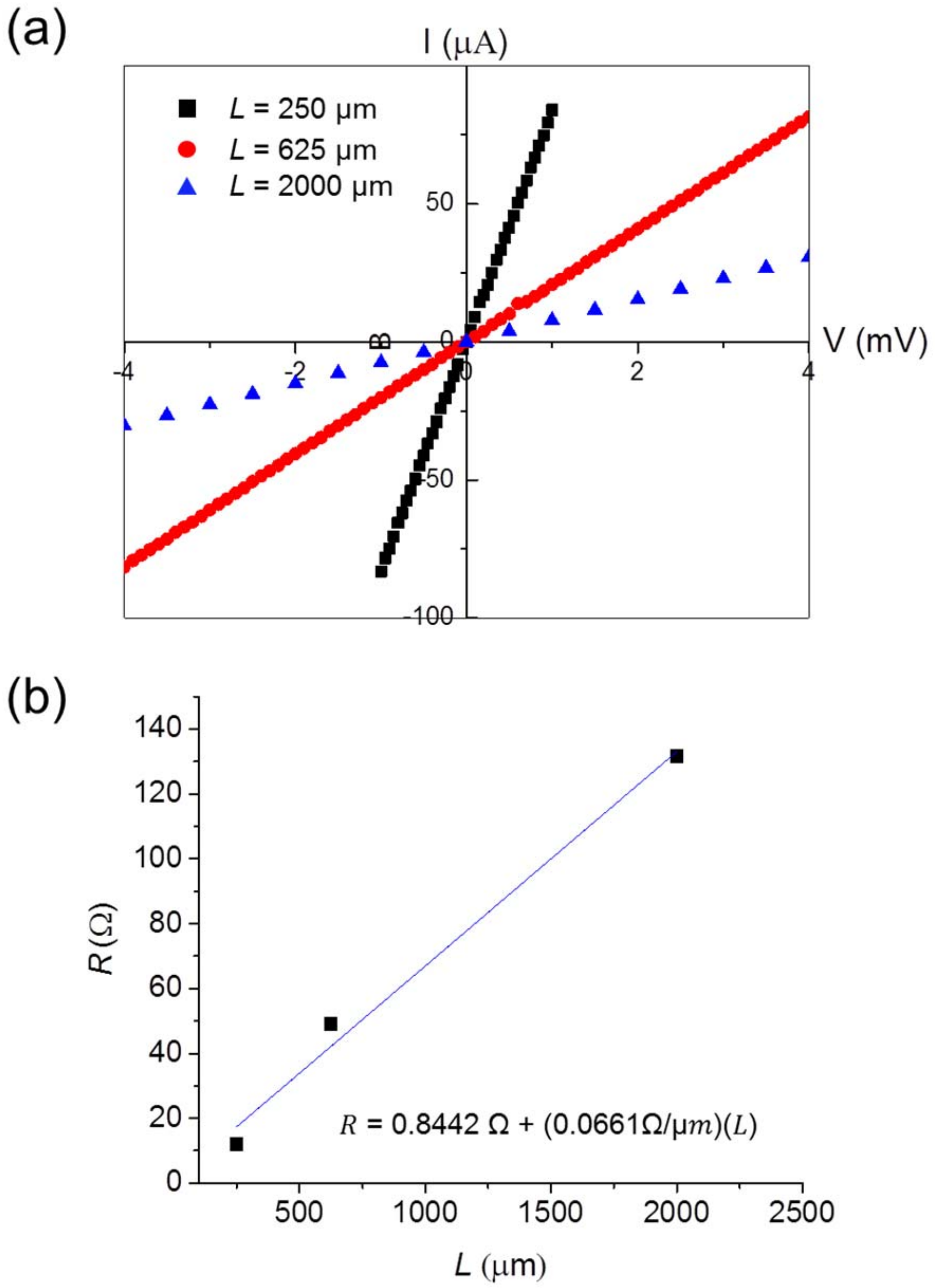

Supplementary Figure S3. (a) Current-voltage (I-V) plots of as-deposited TFMG/TE sample with different contact electrode spacings (*L*) measured at room temperature. (b) Plot of the measured total resistance (*R*) as a function of the contact electrode spacing of as-deposited TFMG/TE sample.

Supplementary Table S1. Electrical properties including sheet resistance (R_s), resistivity (ρ), contact resistance (R_c), transfer length (L_T) and specific contact resistivity (ρ_c) of TFMG/Si, Ni/Si, and TFMG/TE samples.

Sample	Sheet Resistance[1]		Resistivity[1]
	R_s (Ω / □)		ρ(Ω m)
200-nm-TFMG on Si(100)	11.6		2.33×10^{-6}
200-nm-Ni on Si(100)	1.65		3.31×10^{-7}
	Contact Resistivity[2]		
TFMG on Se-doped $AgSbTe_2$	R_c (Ω)	L_T (μm)	ρ_c (Ω m^2)
	0.422	6.385	1.62×10^{-9}

1) The sheet resistance and the resistivity were measured using a resistivity meter.

2) The contact resistance and specific contact resistance was measured based on a transmission line measurement (TLM) technique using the semiconductor parameter analyzer.
